# Supplementary figures and images for: Phenotypic Variability Correlates with Clinical Outcome in Cryptococcus Isolates Obtained from Botswanan HIV/AIDS Patients
Source: mBio. 2018 Oct 23;9(5):e02016-18. doi: 10.1128/mBio.02016-18 (PMC6199498; doi:10.1128/mBio.02016-18)

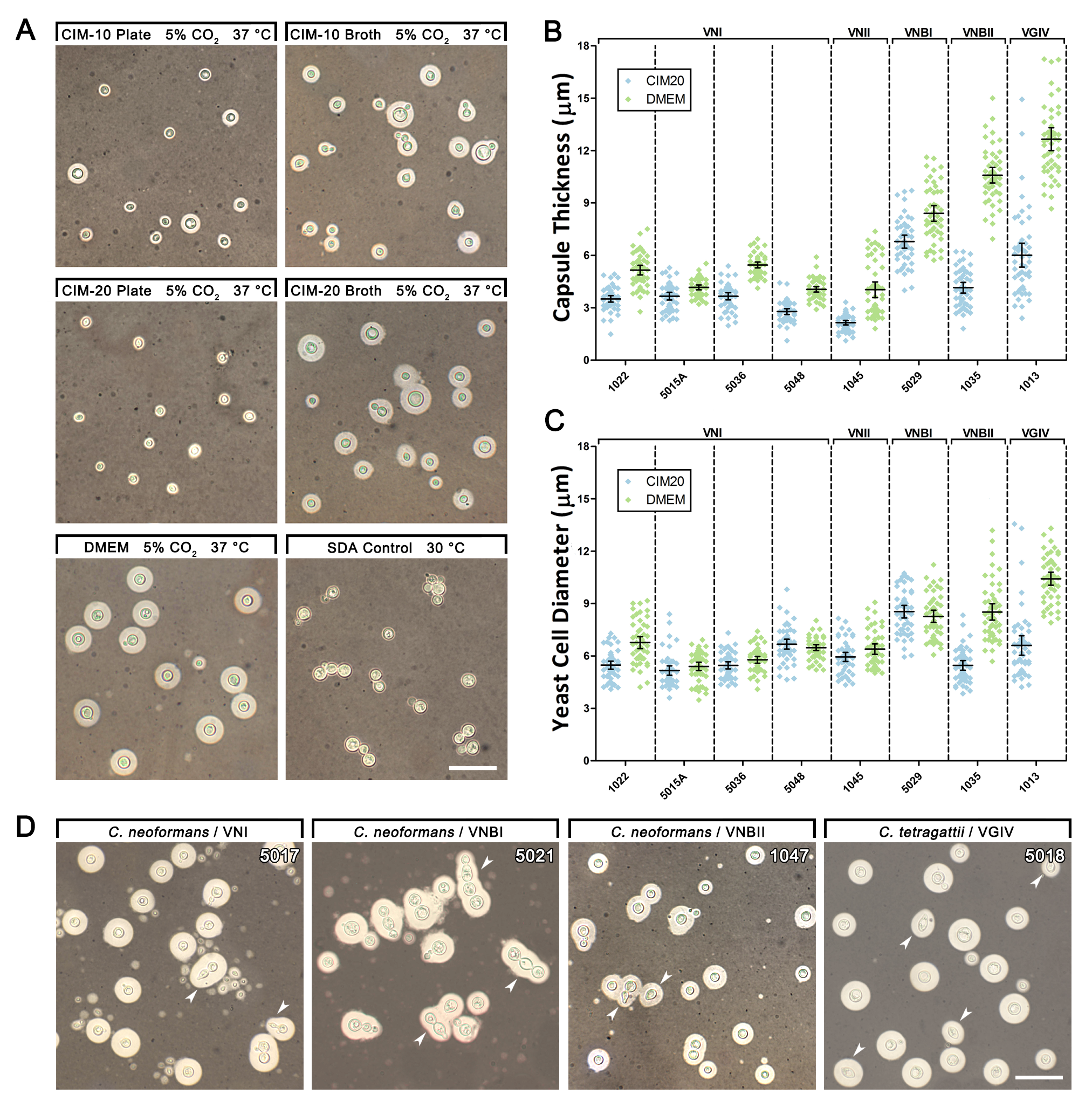

Supplement: FIG S1 [file mbo005184125sf1.tif]
